# Chemical Characterization and Differential Lipid-Modulating Effects of Selected Plant Extracts from Côa Valley (Portugal) in a Cell Model for Liver Steatosis

**DOI:** 10.3390/ph18010039

**Published:** 2025-01-01

**Authors:** Ricardo Amorim, Mário Pedro Marques, Catarina Melim, Carla Varela, Vilma A. Sardão, José Teixeira, Maria Inês Dias, Lillian Barros, Paulo J. Oliveira, Célia Cabral

**Affiliations:** 1 Clinic Academic Center of Coimbra (CACC), Faculty of Medicine, Coimbra Institute for Clinical and Biomedical Research (iCBR), University of Coimbra, 3000-548 Coimbra, Portugal; ricardo.amorim@i3s.up.pt (R.A.); mpedromarques1995@gmail.com (M.P.M.); catarina_melim@hotmail.com (C.M.); carlalvarela@gmail.com (C.V.); 2 Center for Innovative Biomedicine and Biotechnology (CIBB), University of Coimbra, 3000-548 Coimbra, Portugal; vimarisa@ci.uc.pt (V.A.S.); jose.teixeira@uc.pt (J.T.); pauloliv@ci.uc.pt (P.J.O.); 3 Chemical Engineering and Renewable Resources for Sustainability (CERES), Department of Chemical Engineering, University of Coimbra, Pólo II, R. Silvio Lima, 3030-790 Coimbra, Portugal; 4 Multidisciplinary Institute of Aging, University of Coimbra, 3004-504 Coimbra, Portugal; 5 Center for Neuroscience and Cell Biology, University of Coimbra, 3004-504 Coimbra, Portugal; 6 Mountain Research Centre (CIMO), Polytechnic Institute of Bragança (IPB), Campus Santa Apolónia, 5300-253 Bragança, Portugal; maria.ines@ipb.pt (M.I.D.); lillian@ipb.pt (L.B.); 7 Associate Laboratory for Sustainability and Technology in Mountains Regions (SusTEC), Polytechnic Institute of Bragança (IPB), Campus de Santa Apolónia, 5300-253 Bragança, Portugal; 8 Center for Functional Ecology, Department of Life Sciences, University of Coimbra, Calçada Martim de Freitas, 3000-456 Coimbra, Portugal

**Keywords:** Côa Valley (Portugal), plant extracts, *Equisetum ramosissimum* Desf., MASLD, lipid-lowering effect

## Abstract

Background/Objectives: Côa Valley, located in the northeast of Portugal, harbors more than 500 medicinal plant species. Among them, four species stand out due to their traditional uses: *Equisetum ramosissimum* Desf. (hemorrhages, urethritis, hepatitis), *Rumex scutatus* L. subsp. *induratus* (Boiss. and Reut.) Malag. (inflammation, constipation), *Geranium purpureum* Vill., and *Geranium lucidum* L. (pain relief, gastric issues). Given their rich ethnomedicinal history, we evaluated their protective effects on an in vitro model of metabolic dysfunction-associated steatotic liver disease (MASLD). Methods: Decoction (D) and hydroalcoholic (EtOH80%) extracts were prepared and chemically characterized. Their safety profile and effects on lipid accumulation were assessed in palmitic acid (PA)-treated HepG2 cells using resazurin, sulforhodamine B, and Nile Red assays. Results: Chemical analysis revealed diverse phenolic compounds, particularly kaempferol derivatives in *E. ramosissimum*. All extracts showed minimal cytotoxicity at 25–50 µg/mL. At 100 µg/mL, only *E. ramosissimum* extracts maintained high cell viability. In the lipotoxicity model, *E. ramosissimum* decoction demonstrated the most potent effect, significantly reducing PA-induced neutral lipid accumulation in a dose-dependent manner, while other extracts showed varying degrees of activity. Conclusions: These findings highlight *E. ramosissimum’s* decoction, rich in kaempferol derivatives, as particularly effective in reducing lipid accumulation in this MASLD cell model while also providing a comprehensive characterization of traditionally used plants from the Côa Valley region.

## 1. Introduction

Located on the final stretch of the Côa river in the Alto Douro region just north of Portugal, the Côa Valley Archaeological Park is home to over 80 paleolithic rock art sites and more than 1200 decorated rocks. As a result, this unique open-air Palaeolithic rock art area has been recognized as a World Heritage Site by UNESCO since 1998 [1]. Notwithstanding, the Côa Valley, which has a typically hot and dry Mediterranean microclimate, features over 500 plant species with potential medicinal properties. Owing to exposure to adverse environmental conditions, which encompass water scarcity, high temperatures, and intense solar radiation, plants inhabiting this territory are anticipated to synthesize a diverse array of phytochemicals, including polyphenols [2], which might be useful in the management and prevention of oxidative-based diseases like MASLD [3].

**Figure 1:**
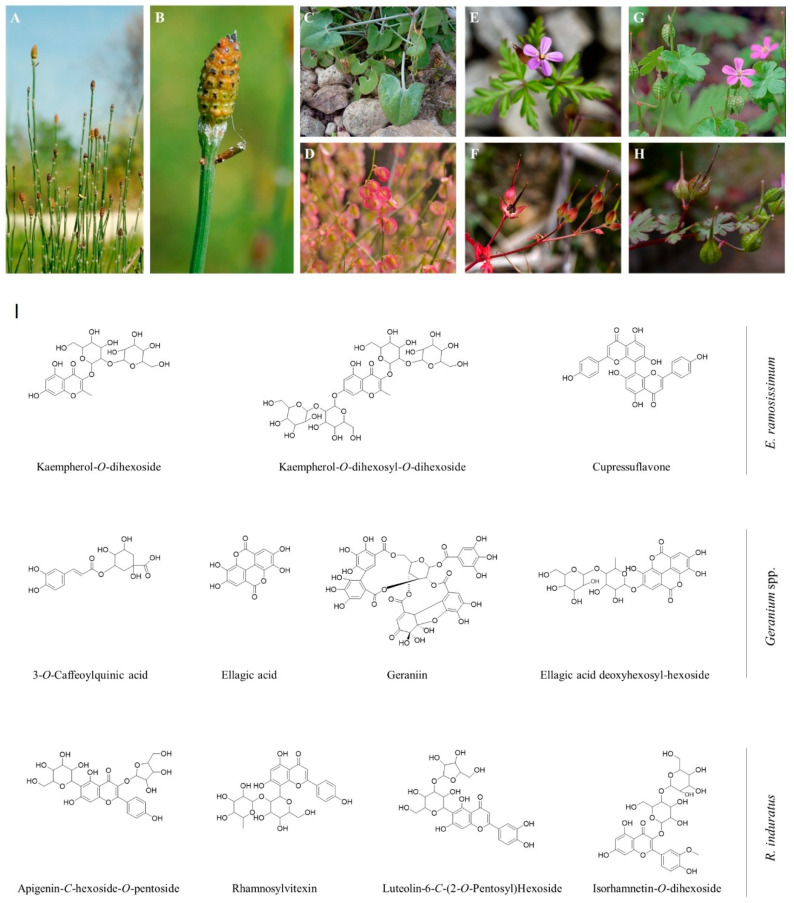
General aspect (**A**) and strobilus, cone-like structure that produces spores at the tips of a stem (**B**) of *E. ramosissimum*. Edible leaves (**C**) and pink-colored fruits (**D**) of *R. induratus*. Close-up on the flower, leaves (**E**), and ripen fruits (**F**) of *G. purpureum*. Flowers, leaves (**G**), and immature fruits of *G. lucidum* (**H**). Images (**A**–**D**,**G**,**H**) were obtained and adapted from [28]. Images (**E**,**F**) were obtained and adapted from [29]. (**I**) Chemical structures of the most relevant phenolic compounds identified through HPLC–DAD–ESI/MS in *E. ramosissimum, Geranium* spp., and *R. induratus* from Côa Valley (Portugal). Chemical structures were designed in the *ChemDraw* Software v.14.0.

Among them, *E. ramosissimum* Desf., an Equisetaceae member (Figure 1A,B), *R. scutatus* L. subsp. *induratus* (Boiss. and Reut.) Malag. from the Polygonaceae family (Figure 1C,D), as well as *G. purpureum* Vill. (Figure 1E,F) and *G. lucidum* L. (Figure 1G,H), both belonging to the Geraniaceae family, are promising medicinal plant species found in this typically dry landscape. Also, *E. ramosissimum*, best known as ramose scouring rush, is the most widely distributed *Equisetum* species, being found in the European, Asian, and African continents, as it can thrive in multiple types of habitats [4,5]. This plant has been used to treat jaundice, hepatitis, hemorrhages, and urethritis [6]. The *Geranium* genus is composed of over 250 species with annual/perennial flowering plants largely found in the northern hemisphere mountainous regions [7]. *G. lucidum* is a small-scaled annual flowering plant with shiny bright green leaves, hence its common names of shiny geranium or shining crane’s bill [8]. *G. purpureum*, also known as little robin, is a plant of spontaneous growth in the Mediterranean and Sub-Mediterranean regions, typically in dry and open spaces [9]. Interestingly, essential oils of these plant species have an intense use in perfumery. Regarding traditional medicine, plants from the genus *Geranium* have been used in the treatment of gastric ailments, inflammatory disorders, hemorrhages, gall bladder, and fever [10]. *R. induratus*, commonly known as buckler sorrel or French sorrel, is a native plant to the Iberian Peninsula that usually grows in stone-filled and dry areas [11,12]. The leaves of the plant are consumed in their raw form, and their sharp, tangy flavor is typically enjoyed in salads or with mashed potatoes seasoned with olive oil [11]. However, it remains underutilized as part of a healthy and balanced dietary regime, as the general population is seldom aware of its potential for consumption, and the plant’s commercialization and distribution are sporadic in local markets [11,13]. The genus *Rumex*, and particularly *R. induratus*, has a wide range of uses in traditional medicine as an antimicrobial, antiviral, anti-inflammatory, and laxative [14,15].

In this study, our objective was to investigate the polyphenol content of four medicinal plant species growing in the Côa Valley region (Portugal) and evaluate their protective effects on a cell model of steatosis observed during MASLD. This is a burgeoning global public health concern, encompassing a spectrum of liver-affecting diseases, ranging from simple steatosis to metabolic dysfunction-associated steatohepatitis, cirrhosis, and eventually hepatocellular carcinoma [16,17]. The pathophysiology of MASLD is multifactorial and markedly associated with genetic, epigenetic, and environmental factors that lead to the accumulation of fat in the liver, culminating in its inflammation and fibrosis [18,19]. MASLD is characterized by several parallel “multihits” that include oxidative stress, generally considered a crucial contributor to liver injury and MASLD pathology [18,20]. Indeed, reactive oxygen species (ROS) cause hepatocellular injury by inhibiting mitochondrial respiratory chain enzymes and inactivating membrane sodium channels and glyceraldehyde-3-phosphate dehydrogenase [21,22]. Oxidative stress also enhances lipid accumulation and cytokine production and intensifies lipid peroxidation, originating oxidized phospholipids and reactive aldehydes that directly induce hepatic inflammation [23,24]. Currently, there are no effective treatments approved for MASLD, with conventional recommendations primarily emphasizing healthy lifestyle modifications and dietary habits for individuals affected by the condition. Polyphenols have become a focal point of extensive research as potential therapeutic agents for the treatment of MASLD [25,26]. These antioxidant compounds are widely found in the chemical composition of several plants and were shown to prevent oxidative stress and alleviate insulin resistance [25,27]. Henceforward, two common extraction methods were employed: ethanol (alcohol), preferred for its ability to extract diverse bioactive compounds, particularly polyphenols, flavonoids, and alkaloids, relevant for conditions like MASLD due to their antioxidant properties. Ethanol at a concentration of 80% (80% ethanol and 20% water) is often preferred because it strikes a balance between extracting a wide range of compounds, including both polar and non-polar constituents. Decoction, a boiling plant material in water, is known for extracting water-soluble compounds such as polysaccharides and saponins, commonly found in traditional medicine. These methods provide insight into potential therapeutic effects, capturing a wide spectrum of bioactive constituents essential for managing conditions like MASLD.

In this work, decoction and EtOH80% extracts of *E. ramosissimum*, *G. lucidum*, *G. purpureum*, and *R. induratus* were prepared and chemically characterized through high-performance liquid chromatography coupled to photodiode array detection and electrospray ionization tandem mass spectrometry (HPLC-DAD-ESI/MS^n^). The cytotoxicity of extracts was determined on human hepatocellular carcinoma (HepG2) cells. Afterward, the extracts were tested in an in vitro cell model of palmitic acid-induced lipotoxicity.

## 2. Results and Discussion

### 2.1. Extract Composition

Different families of phenolic compounds were tentatively identified in the four samples studied. A total of fifty-two phenolic compounds were found, twenty-four in *G. Lucidum* and *G. purpureum* (Table 1), eleven in *E. ramosissimum* (Table 2), and seventeen in *R. induratus* (Table 3), divided into phenolic acids (chlorogenic, caffeic, ferulic, and p-coumaric acid derivatives), ellagic acid derivatives, and flavonoids (O- and C-glycosylated derivatives). The chemical structures of relevant phenolic compounds identified in the studied species were drawn using the ChemDraw Software (Figure 1). The phenolic profile of all species examined in this study has not been previously established by other authors. Only the profile of plants within the same genus is currently available. The identification of phenolic compounds, as described below, relied on comparisons with standards existing in the laboratory, the pertinent literature related to the genus of the studied plants, and additional references describing specific compounds.

#### 2.1.1. Phenolic Acids

Research efforts focusing on the activity of phenolic acids have demonstrated several health benefits, including antioxidant and anti-inflammatory activities and as an antimicrobial agent, and have been linked to the prevention of several cancers and pathologies, like MASLD [27,30,31]. Eleven phenolic acids were found in the four samples studied, mainly chlorogenic, caffeic, ferulic, and *p*-coumaric acid derivatives. Peaks 1^g^ ([M-H]^−^ at *m/z* 353) and 10^e^ ([M-H]^−^ at *m/z* 193), named 3-*O*-caffeoylquinic acid and ferulic acid, respectively, were identified by comparing the retention time, UV, and mass spectra with those of the available standard compounds. The presence of caffeoylquinic acid derivatives in *Geranium* species was already previously reported by [32,33] in *Geranium robertianum* L. and *Geranium molle* L., while ferulic acid was also reported in *Equisetum hyemale* L. extracts by [34].

Glycosylated phenolic acids were also detected in *E. ramosissimum* and *R. induratus* samples. Peak 1^r^ with a protonated ion [M-H]^−^ at *m/z* 341, presented an MS^2^ response characteristic to the caffeic acid molecule (*m/z* at 179 and 135), which corresponded to the loss of the hexosyl moiety (162 u), being tentatively identified as caffeic acid hexoside. The same pattern was observed for peaks 4^e^, 6^e^, 7^r^, and 8^r^ ([M-H]^−^ at *m/z* 355), in which the loss of the hexosyl moiety in the transition to the MS^2^ responses (*m/z* at 193 and 178, ferulic acid molecule) was observed, being all tentatively identified as ferulic acid hexoside. Two glycosylated *p*-coumaric acid derivatives were also found in *R. induratus* samples, peaks 5^r^ and 6^r^ ([M-H]^−^ at *m/z* 325), which, due to the close retention times, were considered *cis* and *trans* isomers, respectively. Finally, peaks 4^g^ ([M-H]^−^ at *m/z* 367) and 3^g^ ([M-H]^−^ at *m/z* 337), tentatively identified as 3-*O*-feruloylquinic and 3-*O*-*p*-coumaroylquinic acid, respectively, were tentatively identified by the previous reported in *Ilex paraguariensis* by [35].

#### 2.1.2. Ellagic Acid Derivatives

Ellagic acid derivatives were only found in *Geranium* spp. samples. The presence of this type of compound has been extensively studied and described by other authors [32,33,36] as the main and the most significant molecule in this genus. Peak 14^g^ was identified as ellagic acid by comparing it with the available standard compound protonated ion ([M-H]^−^ at *m/z* 301) but, above all, with the characteristic UV spectra at 280 nm.

Peaks 5^g^ and 6^g^ ([M-H]^−^ at *m/z* 951), tentatively identified as geraniin and geraniin isomer I, respectively, are the most representative ellagic acid derivatives of this plant genus [32,33,36]. Geraniin demonstrates a remarkable array of health-promoting attributes, including potent antioxidant, antimicrobial, anticancer, cytoprotective, and immune-modulatory properties [37,38]. Moreover, it exhibits analgesic qualities and shows significant promise in addressing hypertension, cardiovascular diseases, and metabolic dysregulation, making it a versatile candidate for therapeutic applications [37]. It was the major compound found in the two *Geranium* samples, especially in the decoction preparation of *G. purpureum* with 39.89 ± 0.04 mg/g extract, representing 61.25% of the total ellagic acid derivatives amount and 50.69% of the total phenolic compounds found in this sample.

Other derivatives of ellagic acid were identified, albeit in a more modest abundance, with their prevalence surpassing that of other compound families, namely peak 11^g^ that presented a deprotonated ion [M-H]^−^ at *m/z* 433 and a unique MS^2^ fragment at *m/z* 301 (ellagic acid) that corresponded to the loss of a pentosyl unit ([M-H-132]^-^) being tentatively identified as ellagic acid pentoside. Peak 11^g^ ([M-H]^−^ at *m/z* 609) loss 308 u (146 u + 162 u), corresponding to a deoxyhexosyl and hexosyl unit, respectively, being tentatively identified as ellagic acid deoxyhexosyl-hexoside. Peaks 7^g^/8^g^/9^g^/10^g^ ([M-H]^−^ at *m/z* 705) were all tentatively identified as ellagic acid dideoxyhexosyl-hexoside, very similar to peak 11^g^, except for the presence of just one more deoxyhexosyl unit. Finally, peak 2^g^ was tentatively identified as ellagitannin solely for the presence of the 301 unit at MS^2^ and the characteristic UV spectra at 270 nm, not allowing the identification of the type of ellagitannin in concrete.

#### 2.1.3. Flavonoids

The flavonoid family was undoubtedly the most representative in terms of the number of compounds identified, highlighting the presence of derivatives of apigenin, isorhamnetin, kaempferol, luteolin, and quercetin.

Peak 14^r^ ([M-H]^−^ at *m/z* 609) was identified as quercetin-3-*O*-rutinoside by comparison with the available standard compound. A large number of identified flavonoids were those with

- One *O*-glycosylation: luteolin-*O*-hexoside (peaks 18^g^/20^g^);
- Two *O*-glycosylation: kaempferol-*O*-dihexoside (9^e^), isorhamnetin-*O*-dihexoside (16^r^/17^r^), isorhamnetin *O*-deoxyhexosyl-hexoside (peak 19^g^), and luteolin-O-deoxyhexosyl-hexoside (peaks 13^g^/17^g^);
- Three *O*-glycosylation: quercetin-*O*-dihexosyl-*O*-dihexoside (peak 3^e^) and kaempferol-*O*-dihexosyl-*O*-dihexoside (peaks 1^e^/2^e^/8^e^) [39];
- An acetyl linkage with *O*-glycosylation: kaempherol-*O*-acetyl-dihexoside (peak 11^e^) and kaempherol-O-hexosyl-O-acetyl-dihexoside (peak 5^e^);
- Phenolic acids linkage with *O*-glycosylation: isorhamnetin-*O*-caffeoyl-*O*-deoxyhexosyl-dihexoside (peaks 3^r^/4^r^), luteolin galloyl *O*-deoxyhexosyl-hexoside (peak 21^g^), luteolin galloyl *O*-hexoside (peak 23^g^), quercetin galloyl *O*-deoxyhexosyl-hexoside (peak 15^g^), and quercetin galloyl *O*-hexoside (peak 16^g^) [40];
- *C*-glycosylation combined or not with *O*-glycosylation: apigenin 6-*C*-hexosyl-8-*C*-pentoside [peak 9^r^, Ref. [41]], apigenin-*C*-hexoside [peak 15^r^, Ref. [42]], apigenin-*C*-hexoside-*O*-pentoside [peaks 12^r^/13^r^, Ref. [42], luteolin-6-*C*-(2-*O*-pentosyl)hexoside [peaks 10^r^/11^r^, Ref. [43]], rhamnosylvitexin [peak 2^r^, Ref. [44]] and cupressuflavone [peak 7^e^, Ref. [45]].

The extensive array of flavonoids found in the four samples underscores the profound significance of this study. The presence of flavonoids exhibiting diverse degrees of polymerization and associations with various functional groups opens countless potential applications for the EtOH80% extracts and decoction preparation of *G. lucidum*, *G. purpureum*, *E. ramosissimum*, and *R. induratus*.

The glycosylation of flavonoids enhances their solubility and stability compared to aglycones, which is especially important given the role of dietary flavonoids and their glycosides in preventing and treating chronic diseases [46]. In both in vitro and in vivo studies, the impact of glycosylation on flavonoid pharmacokinetics is a subject of increasing interest [47]. *C*-glycosyl flavonoids have shown superior antioxidant and anti-diabetic effects in vitro when compared to their *O*-glycosyl counterparts and aglycones. Notably, in some cases, in vitro de-glycosylation of natural flavonoid glycosides has enhanced antioxidant activity [48]. For flavonoids to be efficiently absorbed by the body, they need to reach the small intestine in their unchanged form. It is noteworthy that most flavonoid glycosides maintain their structural integrity even following cooking processes, displaying resistance to the low pH and digestive enzymes present in the stomach. [49].

Finally, peaks 24^g^ and 22^g^ were tentatively identified as luteolin derivative ([M-H]^−^ at *m/z* 575) and quercetin derivative ([M-H]^−^ at *m/z* 591), respectively, presenting both just one MS^2^ fragment at *m/z* 285 and 301, respectively; however, the units lost between full MS and MS^2^ does not give information to identify the sugar moiety and type of linkage.

### 2.2. Effects of the Different Extracts on Metabolic Activity

HepG2 cells are an ideal in vitro model for studying hepatolipotoxicity, given their strong capability to uptake and store fatty acids. This is facilitated by the presence of lipid-metabolizing enzymes like 3-hydroxy-3-methyl-glutaryl-coenzyme A reductase (HMG-CoA reductase) and triglyceride lipase (H-TGL) [50,51].

**Figure 2:**
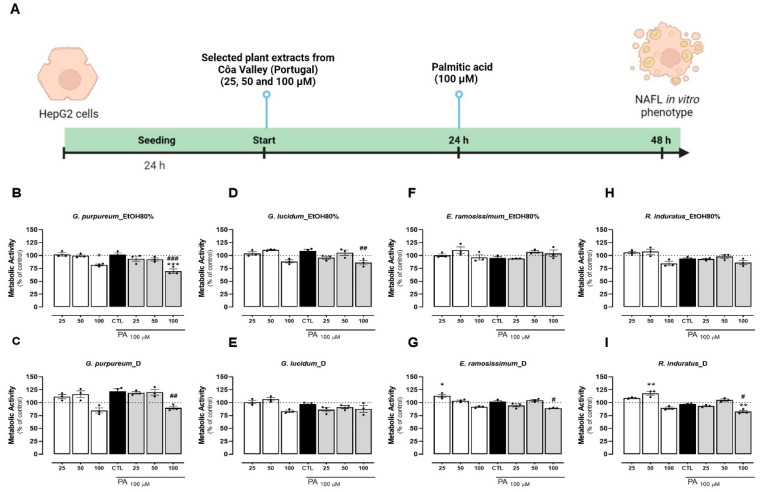
Effects of EtOH80% and decoction extracts of plants originating from the Côa Valley on cell metabolic activity. (**A**) Human cells study experimental timeline. The metabolic activity of HepG2 cells, in percentage of control, following extract incubation in three different concentrations (25, 50, and 100 µg/mL). Each graph contains the results for extract incubation (white bars) and for PA following extract preincubation (grey bars). The black bar represents PA at 100 µM without extract preincubation for comparison purposes. (**B**) EtOH80% and (**C**) D extract of *G. purpureum*, (**D**) EtOH80% and (**E**) D extract of *G. lucidum*, (**F**) EtOH80% and (**G**) D extract of E. ramosissimum, and (**H**) EtOH80% and (**I**) D extract of R. induratus. Statistical significance was compared using two-way ANOVA followed by Tukey post hoc test for multiple comparisons (* *p* < 0.05, ** *p* < 0.01, *** *p* < 0.0005, vs. untreated cells); (^#^ *p* < 0.05, ^##^ *p* < 0.01, ^###^ 0.0005 vs. PA-treated cells).

**Figure 3:**
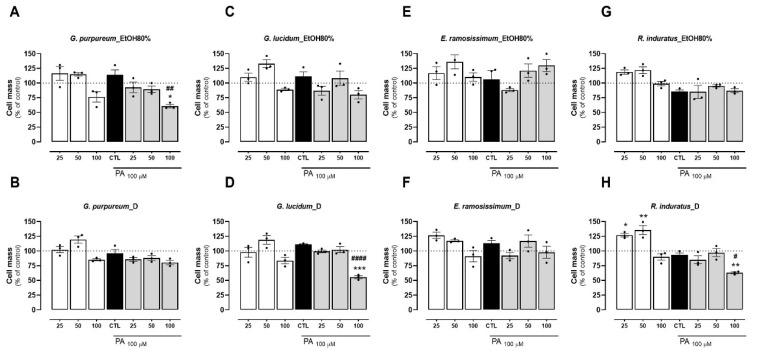
Effects of EtOH80% and decoction (**D**) extracts of plants originating from the Côa Valley on cell mass. The cell mass of HepG2 cells, in percentage of control, following extract incubation in three different concentrations (25, 50, and 100 µg/mL). Each graph contains the results for extract incubation (white bars) and for PA following extract preincubation (grey bars). The black bar represents PA at 100 µM without extract preincubation for comparison purposes. (**A**) EtOH80% and (**B**) D extract of *G. purpureum*, (**C**) EtOH80% and (**D**) D extract of *G. lucidum*, (**E**) EtOH80% and (**F**) D extract of *E. ramosissimum,* (**G**) EtOH80% and (**H**) D extract of *R. induratus*. Statistical significance was compared using two-way ANOVA followed by Tukey post hoc test for multiple comparisons (* *p* < 0.05, ** *p* < 0.01, *** *p* < 0.0005, vs. untreated cells); (^#^ *p* < 0.05, ^##^ *p* < 0.01, ^####^ *p* < 0.0001 vs. PA-treated cells).

To evaluate the hepatoprotective effect of these extracts, it is imperative that the concentration of the plant extract does not induce any toxicity in the cells in the established timeline (Figure 2A). Initially, we examined the cytotoxicity of the extracts on HepG2 cells. Compared to the control group (untreated cells), all extracts exhibited a dose-dependent impact on cellular metabolic activity. Notably, concentrations of 25 and 50 µg/mL resulted in minimal to no reduction in cellular metabolic activity (Figure 2B–I). However, at the highest concentration (100 µg/mL), both extracts of *G. lucidum*, *G. purpureum*, and *R. induratus* exhibited a decrease in metabolic activity compared to untreated cells (Figure 2A–C,H,I). *E. ramosissimum* showed no significant decrease in cell metabolic activity for any of the concentrations tested (Figure 2F–G). Overall, the EtOH80% extracts of *G. purpureum* and *R. induratus* caused the largest decay of cellular metabolic activity at 100 µg/mL, reducing that parameter to 75% and 80%, respectively, when compared to the control (Figure 2B,H). No correlation was found between the extract method and its effects on metabolic activity. From this, we can conclude that all extracts showed no or little cytotoxicity in concentrations up to 50 µg/mL. This observation was also consistent with the positive control, which exhibited no cytotoxic effects up to a concentration of 200 µM (Figure S1A, supplementary information). Concomitantly, the effects of the different extracts were evaluated when incubated with PA. The addition of PA at 100 µM for 24 h did not decrease the metabolic capacity of HepG2 cells, as previously reported (Figure 2B–I) [52,53,54]. Compared with control and PA alone, all extracts presented little to no reduction in the metabolic activity of cells at 25 and 50 µg/mL (Figure 2B–I). Similarly, the highest extract concentration (100 µg/mL), apart from the EtOH80% extract of *E. ramosissimum,* resulted in a reduction (under 20%) in the metabolic activity when compared with control and PA addition, indicating cytotoxicity for the highest extract concentration (Figure 3A–H). Of the four plants tested, both extracts of *E. ramosissimum* were safe to HepG2 cells even for the highest concentration.

Overall, the EtOH80% extract of *G. purpureum* presented the largest decrease in cell metabolic activity, particularly in the highest concentration and following PA incubation (Figure 2B). This finding is in line with what has been previously described for *Geranium* species, which demonstrates an antiproliferative capacity against several cancer cell lines, including for the hepatocellular carcinoma HuH7 cell line [55]. Conversely, the EtOH80% extract of *E. ramosissimum* showed minimal toxicity to HepG2 cells, even at high concentrations (Figure 2F). A dose-dependent decrease in cell metabolic activity was observed for the decoction, which is in agreement with results obtained in human melanoma cells following incubation with an aqueous extract of *E. ramosissimum* (Figure 2G) [6]. Given that the primary compounds identified in both extracts of *E. ramosissimum* in this study are kaempferol derivatives, the safety of these plant extracts on HepG2 cells might be attributed to the documented low toxicity of kaempferol on this cell line. Prior research has demonstrated that kaempferol exhibits minimal toxicity on HepG2 cells, even at concentrations up to 100 µM and during incubation periods of up to 48 h [56].

### 2.3. Effects of Tested Extracts on Cell Mass

HepG2 cells were incubated with decoction and EtOH80% extracts of plants for 24 h at different concentrations (25, 50, and 100 µg/mL). The cell protein, an indirect measurement of cell mass, was further determined by the SRB assay. As observed for the metabolic activity assay, all extracts presented little to no reduction in the cell mass at 25 and 50 µg/mL when compared with the control (untreated cells) (Figure 3A–H). Of the four plants tested, the EtOH80% extract of *E. ramosissimum* did not reduce cell mass even at the highest concentration (Figure 3E). Overall, both extracts of *G. purpureum* at 100 µg/mL resulted in a loss of cell mass (Figure 3A–B). Of all plants, an increase in cell mass was observed at 25 and 50 µg/mL was observed only for *R. induratus* (Figure 3H). Remarkably, a similar trend was observed with silibinin at 100 µM. Notably, the cell mass of HepG2 increased by 15% and 30% at concentrations of 50 and 100 µg/mL of silymarin, respectively (Figure S1B, supplementary information). Among the tested extracts, only the decoctions of *E. ramosissimum* and *R. induratus* caused a statistically significant decrease in cell mass. This may suggest that the aforementioned extracts stimulate the proliferation of HepG2 cells. No correlation was found between the extract type and variation in cell mass.

Subsequently, the impacts of various extracts were assessed during cell incubation with palmitic acid (PA). As observed previously for the metabolic activity assay, all plant extracts caused little to no reduction in cell mass at 25 and 50 µg/mL when compared with the control (BSA) and PA regimens (Figure 3A–H). In contrast, the highest concentration (100 µg/mL) tested for all extracts resulted in a decrease in cell mass of up to 50% of the control value and PA. Notably, the cell mass increase reported for the extracts was not observed following PA addition. This can be due to the toxic action of the lipid that, after being added to cells following a 24 h extract incubation, resulted in cell death. Overall, the EtOH80% extract of *G. lucidum* presented the worst outcome, particularly at the highest concentration and following PA incubation, in which a cell mass decrease close to 50% was observed (Figure 3D). As observed for the metabolic activity, out of the four plants tested, both extraction methods of *E. ramosissimum* showed to be safe to the cells even at the highest concentration tested (Figure 3E–F). As already mentioned above for the cell metabolic activity, the safety of the *E. ramosissimum* extract may be associated with the high kaempferol content of both extracts, which has been described before as presenting little toxicity to HepG2 cells up to 48 h of incubation [56].

### 2.4. Effects of Tested Extracts on Preventing PA-Induced Lipid Accumulation in HepG2 Cells

**Figure 4:**
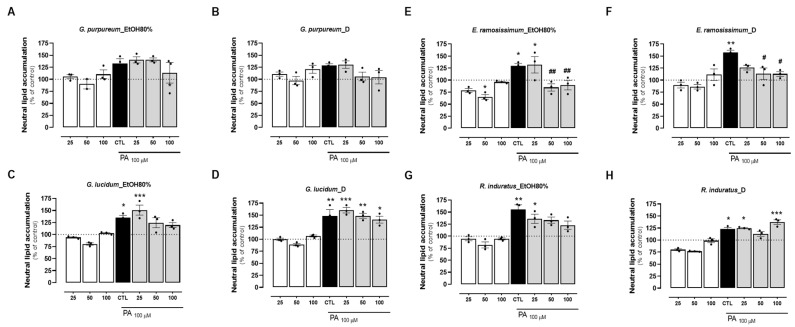
Effects of EtOH80% and decoction extracts of plants originating from the Côa Valley on cell lipid accumulation. The neutral lipid accumulation of HepG2 cells, in percentage of control and normalized for cell mass results, following extract incubation in three different concentrations (25, 50, and 100 µg/mL). Each graph contains the results for extract incubation (white bars) and for PA following extract preincubation (grey bars). The black bar represents PA at 100 µM without extract preincubation for comparison purposes. (**A**) EtOH80% and (**B**) D extract of *G. purpureum*, (**C**) EtOH80% and (**D**) D extract of *G. lucidum*, (**E**) EtOH80% and (**F**) D extract of E. ramosissimum, (**G**) EtOH80% and (**H**) D extract of R. induratus. Statistical significance was compared using two-way ANOVA followed by Tukey post hoc test for multiple comparisons (* *p* < 0.05, ** *p* < 0.01, *** *p* < 0.0005, vs. untreated cells); (^#^ *p* < 0.05, ^##^ *p* < 0.01vs. PA-treated cells).

To investigate cellular lipotoxicity, palmitate (PA) is commonly employed to markedly enhance lipid droplet (LD) formation. This triggers an increase in ROS levels, leading to mitochondrial dysfunction manifested by decreased O_2_ levels and mitochondrial membrane potential (ΔΨm) [16,57,58]. Taking this into account, we sought to investigate the lipid accumulation lowering potential of the same extracts on PA-treated HepG2 cells. PA treatment significantly increased the neutral lipid accumulation in HepG2 up to 50% when compared to the control (BSA) in all groups tested (Figure 4A–H). From the *Geranium* species, the decoction of *G. purpureum* and *G. lucidum* EtOH80% extract, at concentrations of 50 and 100 µg/mL, resulted in a slight reduction in the accumulation of neutral lipids when compared with the PA values, although not reaching statistical significance (Figure 4B,C). Similarly, a slight decrease in the lipid content was measured for the EtOH 80% extract of *R. induratus*, which was also dose-dependent (Figure 4G). Of all the plants, both extracts from *E. ramosissimum* decreased the accumulation of neutral lipids when compared to PA alone (Figure 4E,F). More precisely, the decoction of *E. ramosissimum* significantly reduced the neutral lipid accumulation in a dose-dependent manner to values closer to the control in all concentrations (Figure 4F). Interestingly, for the highest concentration, the extent of the reduction in lipid accumulation observed for this extract was higher than that obtained for the lipid-decreasing compound silibinin (44% vs. 27% reduction compared with PA values, respectively (Figure S1C, supplementary information). This effect may be associated with the anti-lipotoxicity and anti-adipogenic effects of the phenolic compound kaempferol, which was identified in the chemical composition of *E. ramosissimum.* In addition to this work, kaempferol was previously described in the chemical composition of EtOH80% extract of stems from *E. ramosissimum* [59]. This flavonol has been associated with lower lipogenesis by upregulating the expression of Insing-2a, decreasing the phosphorylation of SREBP-1, but increasing GKS-3 phosphorylation. This culminates in the inactivation of SREBP-1, a key lipogenic transcription factor [60,61]. Another paper also described the capacity of kaempferol to decrease lipid accumulation and oxidative stress on HepG2 cells [62]. Our results are also corroborated by recent work performed by other authors [63,64], in which the protective effect of kaempferol on lipid accumulation in HepG2 through activation of the NF-E2–related factor 2 (Nrf2) signaling pathway was reported. Furthermore, kaempferol modulates inflammatory responses through the NF-κB pathway [65], as demonstrated in oleic acid-induced HepG2 cells, thereby inhibiting inflammation and fibrosis in high-fat diet-induced rats [66].

The observed reduction in neutral lipid accumulation suggests potential anti-lipotoxic and anti-adipogenic properties of *E. ramosissimum*. Future research will investigate these mechanisms further and determine their specific contributions to the observed effects. *E. ramosissimum* shows significant promise in the treatment of MASLD, achieving a notable 44% reduction in lipid accumulation. This surpasses or equals the efficacy of the reference compound silibinin and other natural compounds like curcumin [67] and green tea extracts [68]. In addition, *E. ramosissimum* stands out for its exceptional safety at high doses, offering a considerable advantage in therapeutic development. While these findings are promising, they must be interpreted with caution due to the limitations of the in vitro model. Cell culture systems cannot fully replicate the complexity of MASLD in humans, which involves systemic inflammation, metabolic interactions, and the participation of multiple organs. Additionally, the bioavailability and metabolism of plant-derived compounds in vivo may differ significantly from those observed under experimental conditions. These findings represent an encouraging first step and highlight the need for further investigations in more complex biological systems.

## 3. Materials and Methods

### 3.1. Materials

Bovine serum albumin (BSA), Dulbecco’s modified Eagle’s medium (DMEM, D5030), fetal bovine serum (FBS), glucose, glutamine, HEPES, Nile Red, palmitic acid (C16:0) (PA), resazurin, silibinin, sodium pyruvate, and sulforhodamine B (SRB) were purchased from Sigma-Aldrich (St. Louis, MO, USA). Penicillin, streptomycin, and trypsin were purchased from Gibco-Invitrogen (Grand Island, NY, USA). Sodium bicarbonate was purchased from Thermo Scientific (Waltham, MA, USA).

### 3.2. Plant Material

Samples of the plant *E. ramosissimum*, flowering aerial parts of *G. lucidum* and *G. purpureum*, and *R. induratus* in the fruiting phase were collected in May 2021. The four studied plants were collected in the Côa Valley River. Voucher specimens were placed at the Herbarium of the University of Aveiro (AVE), with the numbers AVE251 representing *E. ramosissimum*, AVE256 representing *G. lucidum*, AVE263B representing *G. purpureum*, and AVE254A representing *R. induratus*. After the harvesting process, the plants were dried and kept at room temperature (25 °C) in the dark until extraction.

### 3.3. Extraction Procedures

#### 3.3.1. Decoction Extraction

Initially, water (150 mL) was poured into the dried and powdered plant (5 g). The extraction procedure occurred with magnetic stirring at boiling temperature for 20 min. and shielded from the light with aluminum foil. Once the decoction extract was at room temperature, it was filtered under vacuum with a Büchner funnel and Whatman No. 4 paper, followed by a reduction of its volume using a vacuum rotator. After lyophilization, the decoction residue was obtained. The extract yields were as follows: 21.2% for *E. ramosissimum*, 30.6% for *G. lucidum*, 32.4% for *G. purpureum*, and 3.6% for *R. induratus*.

#### 3.3.2. Hydroalcoholic Extraction

To dried and powdered plant material (5 g), a hydroalcoholic extraction was performed using an ethanol/water (80:20 *v*/*v*) (EtOH80%) solution (125 mL), with magnetic stirring at room temperature for 1 h, and shielded from the light with aluminum foil. After that, the mixture was decanted. To the remaining plant material, another EtOH80% solution (125 mL) was poured, and extraction occurred under the same conditions. This extract was also decanted, and the two extraction solutions combined, and filtered under vacuum with a Büchner funnel and Whatman No. 4 paper. Ethanol was evaporated using a vacuum rotator (Büchi Rotavapor® R-300, Barcelona, Spain) and the obtained aqueous residue was subsequently freeze-dried. The obtained extract yields were as follows: 12.6% for *E. ramosissimum*, 21.4% for *G. lucidum*, 26.4% for *G. purpureum*, and 1.2% for *R*. *induratus*. The obtained lyophilized EtOH80% and decoction extracts were redissolved in water to a final concentration of 10 mg/mL, functioning as stock solutions preserved at −20 °C. These stock solutions were further diluted to different concentrations to be tested in the subsequent assays.

### 3.4. HPLC–DAD–ESI/MS Analysis of Hydroalcoholic and Decoction Extracts

The extracts were analyzed as previously stated by us in [69], with minimal adjustments. Briefly, all extracts were filtered through a 0.22 µm disposable LC filter disk, and the extracts’ samples were analyzed with a Dionex Ultimate 3000 UPLC (Thermo Scientific, San Jose, CA, USA) system equipped with a diode array detector with an electrospray ionization mass detector coupled to it (LC-DAD-ESI/MS^n^). A Waters Spherisorb S3 ODS-2 C18 (3 μm, 4.6 × 150 mm, Waters, Milford, MA, USA), with the column thermostatized at exactly 35 °C, was used to achieve chromatographic separation. Solvents used consist of 0.1% formic acid in water and acetonitrile, using an elution grade for 50 min and re-equilibration of the column for 10 min, with a flow rate of 0.5 mL/min. Double online detection was performed in the DAD (using 280 and 370 nm as preferred wavelengths), as well as in a mass spectrometer (MS) that was connected to an HPLC system through the DAD cell outlet. Detection of MS was carried out using a Linear Ion Trap LTQ XL mass spectrometer (Thermo Finnigan, San Jose, CA, USA) in the negative mode, equipped with an ESI source. Data were obtained with the Xcalibur^®^ (Xcalibur™ 4.3 software) data system (Thermo Finnigan, San Jose, CA, USA). Regarding phenolic compounds, these were identified by comparing their retention times, mass spectra, and UV–Vis with standard compounds when such comparison is available. If not, compounds were identified by comparing the aforementioned data with those reported in the literature. Compound quantification was performed using the following calibration curves: apigenin-6-*C*-glucoside (*y* = 107.025*x* + 61.531, *R*^2^ = 0.999, Limit of detection (LOD) = 0.19 µg/mL; Limit of Quantification (LOQ) = 0.63 µg/mL); apigenin-7-*O*-glucoside (*y* = 10.683*x* − 45.794; *R^2^* = 0.999, LOD = 0.10 μg/mL; and LOQ = 0.53 μg/mL); caffeic acid (*y* = 388.345*x* + 406.369, *R*^2^ = 0.994, LOD = 0.78 μg/mL; LOQ =1.97 μg/mL); chlorogenic acid (*y* = 168.823*x* − 161.172, *R*^2^ = 0.9999, LOD = 0.20 µg/mL; LOQ = 0.68 µg/mL); ferulic acid (*y* = 633.126*x* − 185.462, *R*^2^ = 0.999, LOD = 0.20 μg/mL; 1.01 μg/mL); ellagic acid (*y* = 26.719*x* − 317.255, *R^2^* = 0.999, LOD = 41.20 µg/mL; LOQ = 124.84 µg/mL); *p*-coumaric acid (*y* = 301.950*x* + 6966.7, *R*^2^ = 0.999, LOD = 0.68 μg/mL, LOQ = 1.61 μg/mL); and quercetin-3-*O*-glucoside (*y* = 34.843*x* − 160.173, *R*^2^ = 0.999, LOD = 0.21 µg/mL; LOQ = 0.71 µg/mL). For the compounds without a standard compound, the calibration curve of the most structurally similar compound was used. The obtained results were presented as mg per g of extract.

### 3.5. Cell Culture and Extract Treatment

Human hepatocellular carcinoma (HepG2) cells (Catalogue nº: 85011430, ECACC, Porton Down, UK) were cultured in a 5% CO_2_ atmosphere at 37 °C in low-glucose DMEM supplemented with 5 mM glucose, 1mM sodium pyruvate, 6 mM L-glutamine, 5 mM HEPES, 3.7 g/L sodium bicarbonate, 10% FBS, and 1% antibiotic-antimycotic (penicillin-streptomycin 100x solution) as previously described [70,71]. Cells were seeded at 4.5 × 10^4^ cells/cm^2^ in 96-well plates and grown until 70% confluence. After that, cells were incubated with different plant extracts at different concentrations (50, 100, and 200 µg/mL) for 24 h. Following extract incubation for 24 h, the cells were treated with vehicle BSA (0.1 g/mL) and PA (0.1 mM).

### 3.6. Palmitic Acid/BSA Conjugation

The conjugation of PA with BSA was performed as previously described [70]. Briefly, PA and BSA were prepared using BSA free-fatty acid (Catalogue A6003, Sigma-Aldrich, St. Louis, MO, USA), with a concentration of 0.1 g/mL. PA was combined with BSA at a 1:1 ratio and incubated for 1 h at 37 °C. Additionally, a control solution was prepared using the same proportion of free-fatty acid BSA (0.1 g/mL) diluted with 150 mM NaCl.

### 3.7. Cell Metabolic Activity

HepG2 cells were seeded in 96-well plates and subjected to different treatments. Following incubation, the cell metabolic activity was determined following the resazurin reduction principle [72]. Resazurin (blue, non-fluorescent) is reduced to resorufin (pink, highly fluorescent) by metabolically active cells through mitochondrial enzymes (NADH dehydrogenases), providing a quantitative measure of cell viability and metabolic activity. After discarding the culture medium, cells were incubated for 2 h with 80 µL of culture medium supplemented with 10 µg/mL of resazurin solution. The fluorescence was measured with 570 nm excitation and 600 nm emission wavelengths in a Biotek Cytation 3 reader (Biotek Instruments, Winooski, VT, USA).

### 3.8. Cell Mass

Cells were seeded in a 96-well plate and subjected to different treatments. SRB assay was then performed for cell mass determination based on the measurement of cellular protein content [73]. The Sulforhodamine B (SRB) assay quantifies cellular protein content by binding to basic amino acid residues under mildly acidic conditions. This provides an accurate measure of cell mass since total protein content is directly proportional to cell number. After discarding the culture medium, wells were rinsed with PBS (1X), and cells were fixed by adding 80 µL of 1% acetic acid in 100% methanol solution and kept at −20 °C overnight. Then, the fixation solution was discarded, and the plate was kept in the incubator(NUAIRE NU-4850 CO2 incubator, Artisan Technology Group, Illinois, USA) until completely dry at 37 °C. 50 µL of 0.05% SRB in 1% acetic acid solution was added to the wells and incubated for 1 h at 37 °C. The wells were washed with 1% acetic acid solution to remove unbound SRB solution residues. Following this, 100 µL of Tris (10 mM, pH 10.5) solution was used to dissolve SRB by stirring the plate for 30 min using an orbital shaker. The absorbance was measured at 510 nm and the background measurement at 620 nm at room temperature in a Biotek Cytation 3 reader (Biotek Instruments, Winooski, VT, USA).

### 3.9. Nile Red Staining

Following the different treatments, the neutral lipid accumulation in HepG2 cells was evaluated using the Nile Red assay [74]. After discarding the culture medium, 100 µL of Nile Red solution (freshly diluted from a stock solution of 0.5 mg/mL in acetone at 1:200 in medium without FBS) was added to each well and incubated for 2 h protected from the light at 37 °C. Then, the Nile Red solution was removed, and the cells were washed with PBS (1X) twice. The lipid content in the wells was measured in fluorescence with 520 nm excitation and 620 nm emission in a Biotek Cytation 3 reader (Biotek Instruments, Winooski, VT, USA). The results were normalized for the cell mass content of the wells using the SRB assay [73].

### 3.10. Statistic Analysis

Data obtained from cell experiments were analyzed using GraphPad Prism 8.02 software (GraphPad Software, Inc). Data from multiple experiments is presented as the mean ± standard error of the mean (SEM). Statistical significance was assessed using two-way ANOVA followed by the Tukey post hoc test for multiple comparisons to compare different extract concentrations with the control. Statistical significance was accepted with * *p* < 0.05, ** *p* < 0.01, *** *p* < 0.0005, **** *p* < 0.0001 for comparisons between extract concentrations vs. control, and ^#^ *p* < 0.05, ^##^ *p* < 0.01, ^###^ *p* < 0.0005, ^####^ *p* < 0.0001 for comparisons between extract concentrations vs. PA.

## 4. Conclusions

The in-depth chemical analysis of EtOH80% extracts and decoctions from *G. lucidum*, *G. purpureum*, *E. ramosissimum*, and *R. induratus* revealed a rich array of phenolic components, including flavonoids, phenolic acids, and ellagic acid derivatives. These compounds contribute to the pharmacological potential of the extracts, known for their diverse health benefits such as anti-inflammatory, antioxidant, antibacterial, and anticancer effects. The presence of glycosylated flavonoids, particularly in the EtOH80% extracts, highlights their enhanced stability and solubility, potentially improving their bioavailability and medicinal effectiveness. In fact, *C*-glycosyl flavonoids have demonstrated significantly stronger antioxidant and anti-diabetic actions compared to their *O*-glycosyl counterparts, underlining the importance of structural modifications in enhancing biological activity. Biological assessment using HepG2 cells indicated minimal cytotoxicity of the extracts at concentrations up to 50 µg/mL, with no significant impact on cell metabolic activity or mass. However, at higher concentrations (100 µg/mL), some extracts, particularly those of *G. purpureum* and *R. induratus*, exhibited cytotoxic effects. Interestingly, extracts of *E. ramosissimum* showed minimal toxicity even at the highest concentration, possibly due to the presence of kaempferol derivatives known for their low toxicity on HepG2 cells. Furthermore, the extracts, especially the decoction of *E. ramosissimum*, demonstrated promising effects in preventing PA-induced lipid accumulation in HepG2 cells.

## Figures and Tables

**Table 1:** Peaks, retention times (RT), wavelengths of maximum absorption (λmax), mass spectral data, tentative identification, and quantification (mg/g extract) of the phenolic compounds present in the hydroalcoholic (EtOH 80%) and decoction extracts of *G. lucidum* and *G. purpureum* (Mean ± SD). Major compounds identified are highlighted in bold and shaded in gray.

Peak	RT (min)	λ_max_ (nm)	[M-H]^−^ (*m/z*)	MS^n^ (*m/z*)	Tentative Identification	Quantification (mg/g)			
						*G. lucidum*		*G. purpureum*	
						EtOH 80%	Decoction	EtOH 80%	Decoction
**1^g^**	**4.94**	**321.00**	**353**	**MS^2^: ** **191(100), 179(82), 135(12)**	**3-*O*-caffeoylquinic Acid**	**10.14 ± 0.037**	**9.66 ± 0.012**	**2.33 ± 0.026**	**9.97 ± 0.020**
**2^g^**	**5.75**	**270**	**799**	**MS^2^: ** **301(100)**	**Ellagitannin**	**2.21 ± 0.019**	**3.09 ± 0.032**	**1.93 ± 0.004**	**2.87 ± 0.012**
**3^g^**	6.59	324	337	MS^2^: 191(12), 163(100), 119(23)	3-*O*-*p*-Coumaroylquinic Acid	0.70 ± 0.002	0.84 ± 0.019	1.45 ± 0.033	1.29 ± 0.018
**4^g^**	7.63	326	367	MS^2^: 193(100), 191(16), 173(14), 149(25)	3-*O*-Feruloylquinic Acid	0.52 ± 0.006	0.65 ± 0.003	0.21 ± 0.001	0.73 ± 0.012
**5^g^**	**11.06**	**277**	**951**	**MS^2^: ** **933(100), 613(4), 462(6), 301(8)**	**Geraniin Isomer I**	**14.32 ± 0.008**	**4.20 ± 0.071**	**7.47 ± 0.036**	**39.89 ± 0.040**
**6^g^**	12.15	277	951	MS^2^: 933(100), 613(4), 462(6), 301(8)	Geraniin Isomer II	n.d.	n.d.	n.d.	4.22 ± 0.015
**7^g^**	14.55	281	755	MS^2^: 301(100)	Ellagic Acid dideoxyhexosyl-hexoside	1.48 ± 0.006	1.50 ± 0.003	n.d.	n.d.
**8^g^**	**15.84**	**280**	**609**	**MS^2^:** **301(100)**	**Ellagic Acid deoxyhexosyl-hexoside Isomer I**	**1.75 ± 0.005**	**1.36 ± 0.001**	**1.29 ± 0.005**	**1.89 ± 0.026**
**9^g^**	**16.17**	**280**	**609**	**MS^2^:** **301(100)**	**Ellagic Acid deoxyhexosyl-hexoside Isomer II**	**2.33 ± 0.048**	**1.65 ± 0.002**	**1.29 ± 0.001**	**1.94 ± 0.007**
**10^g^**	**16.54**	**280**	**609**	**MS^2^:** **301(100)**	**Ellagic Acid deoxyhexosyl-hexoside Isomer III**	**2.23 ± 0.018**	**1.68 ± 0.015**	**1.34 ± 0.001**	**1.90 ± 0.000**
**11^g^**	**17.06**	**281**	**433**	**MS^2^:** **301(100)**	**Ellagic acid pentoside**	**2.12 ± 0.004**	**1.79 ± 0.006**	**1.66 ± 0.002**	**3.09 ± 0.002**
**12^g^**	17.76	279	609	MS^2^: 301(100)	Ellagic acid deoxyhexosyl-hexoside Isomer IV	1.81 ± 0.021	1.59 ± 0.008	0.54 ± 0.008	4.02 ± 0.089
**13^g^**	18.56	348	593	MS^2^: 285(100)	Luteolin-*O*-deoxyhexosyl-hexoside Isomer I	1.63 ± 0.019	0.95 ± 0.005	n.d.	0.79 ± 0.006
**14^g^**	**19.04**	**280**	**301**	**-**	**Ellagic Acid**	**3.42 ± 0.000**	**7.99 ± 0.004**	**2.13 ± 0.009**	**5.32 ± 0.139**
**15^g^**	19.83	361	761	MS^2^: 609(12), 301(100), 151(12)	Quercetin galloyl *O*-deoxyhexosyl-hexoside	0.84 ± 0.014	1.11 ± 0.001	n.d.	n.d.
**16^g^**	20.33	357	615	MS^2^: 463(12), 301(100)	Quercetin galloyl *O*-hexoside	0.74 ± 0.011	0.74 ± 0.012	n.d.	n.d.
**17^g^**	21.08	349	593	MS^2^: 285(100)	Luteolin-*O*-deoxyhexosyl-hexoside Isomer II	0.81 ± 0.005	0.73 ± 0.004	0.52 ± 0.001	0.76 ± 0.008
**18^g^**	21.38	348	447	MS^2^: 285(100)	Luteolin-*O*-hexoside Isomer I	0.96 ± 0.009	0.71 ± 0.010	1.00 ± 0.014	n.d.
**19^g^**	22.03	353	623	MS^2^: 315(100)	Isorhamnetin *O*-deoxyhexosyl-hexoside	0.63 ± 0.008	0.59 ± 0.005	n.d.	n.d.
**20^g^**	22.59	348	447	MS^2^: 285(100)	Luteolin-*O*-hexoside Isomer II	1.00 ± 0.014	0.70 ± 0.004	n.d.	n.d.
**21^g^**	23.11	353	745	MS^2^: 593(12), 459(89), 285(100)	Luteolin galloyl *O*-deoxyhexosyl-hexoside	0.90 ± 0.005	0.61 ± 0.007	0.90 ± 0.005	n.d.
**22^g^**	23.5	353	591	MS^2^: 301(100)	Quercetin derivative	0.83 ± 0.013	0.54 ± 0.002	0.83 ± 0.013	n.d.
**23^g^**	24.27	351	599	MS^2^: 285(100)	Luteolin galloyl *O*-hexoside	0.97 ± 0.007	0.58 ± 0.003	0.97 ± 0.007	n.d.
**24^g^**	26.79	349	575	MS^2^: 285(100)	Luteolin derivative	1.04 ± 0.010	0.59 ± 0.003	1.04 ± 0.010	n.d.
					Total phenolic acids	11.36 ± 0.041	11.14 ± 0.004	3.99 ± 0.060	11.99 ± 0.009
					Total ellagic derivatives	31.67 ± 0.087	24.86 ± 0.087	18.44 ± 0.034	65.13 ± 0.128
					Total flavonoids	10.35 ± 0.012	7.85 ± 0.006	5.8 ± 0.007	1.56 ± 0.002
					Total phenolic compounds	53.38 ± 0.117	43.85 ± 0.085	28.22 ± 0.019	78.69 ± 0.138

Rt: Retention time in minutes; λ_max_: wavelength (nm) of maximum absorption in the UV–visible region; [M-H]^−^: deprotonated ion (negative ion mode) (*m/z*); MS^n^ fragment ions generated in MS^2^ and/or MS^3^ spectra (*m/z*) and relative abundance in brackets, n.d.: not detected. Standard calibration curves used for quantification: chlorogenic acid (*y* = 168.823*x* − 161.172, *R^2^* = 0.999, LOD (limit of detection) = 0.20 µg/mL; LOQ (limit of quantification) = 0.68 µg/mL, peak 1); ferulic acid (*y* = 633.126*x* − 185.462, *R^2^* = 0.999, LOD = 0.20 μg/mL; 1.01 μg/mL, peak 4); *p*-coumaric acid (*y* = 301.950*x* + 6966.7, *R^2^* = 0.9999, LOD = 0.68 μg/mL and LOQ = 1.61 μg/mL, peak 3); ellagic acid (*y* = 26.719*x* − 317.255, *R^2^* = 0.999, LOD = 41.20 µg/mL; LOQ = 124.84 µg/mL, peaks 2, 5–12); and quercetin-3-*O*-glucoside (*y* = 34.843*x* − 160.173, *R^2^* = 0.999, LOD = 0.21 µg/mL; LOQ = 0.71 µg/mL, peaks 13, 15–24).

**Table 2:** Peaks, retention times (RT), wavelengths of maximum absorption in the visible region (λmax), mass spectral data, tentative identification, and quantification (mg/g extract) of the phenolic compounds present in the hydroalcoholic (EtOH 80%) and decoction extracts of *E. ramosissimum* (Mean ± SD). Major compounds identified are highlighted in bold and shaded in gray.

Peak	RT (min)	λ_max_ (nm)	[M-H]^−^ (*m/z*)	MS^n^ (*m/z*)	Tentative Identification	Quantification (mg/g Extract)	
						EtOH 80%	Decoction
**1^e^**	**5.08**	**246,271sh,352**	**771**	**MS^2^: 609(100). MS^3^: 429(100), 285(53)**	**Kaempherol-*O*-dihexosyl-*O*-dihexoside Isomer I**	**5.64 ± 0.005**	**4.08 ± 0.007**
**2^e^**	**5.93**	**246,271sh,352**	**771**	**MS^2^: 609(100). MS^3^: 429(100), 285(35)**	**Kaempherol-*O*-dihexosyl-*O*-dihexoside Isomer II**	**1.24 ± 0.008**	**0.93 ± 0.001**
**3^e^**	6.36	264,301,328	787	MS^2^: 625(100). MS^3^: 463(100), 301(79)	Quercetin-*O*-dihexosyl-*O*-dihexoside	0.76 ± 0.012	0.76 ± 0.001
**4^e^**	6.70	284.00	355	MS^2^: 193(100), 178(18)	Ferulic acid hexoside Isomer I	0.31 ± 0.005	0.22 ± 0.004
**5^e^**	7.83	266,248	813	MS^2^: 651(100), 285(34)	Kaempherol-*O*-hexosyl-*O*-acetyl-dihexoside	0.66 ± 0.004	0.60 ± 0.001
**6^e^**	8.25	284.00	355	MS^2^: 193(100), 178(23)	Ferulic acid hexoside Isomer II	0.59 ± 0.010	0.41 ± 0.006
**7^e^**	**10.68**	**266,307,327**	**537**	**MS^2^: 375(100), 195(34)**	**Cupressuflavone**	**2.21 ± 0.007**	**2.00 ± 0.004**
**8^e^**	**15.11**	**246,271sh,352**	**771**	**MS^2^: 609(100). MS^3^: 429(100), 285(98)**	**Kaempherol-*O*-dihexosyl-*O*-dihexoside Isomer III**	**0.97 ± 0.006**	**0.75 ± 0.008**
**9^e^**	**16.57**	**347.00**	**609**	**MS^2^: 285(100)**	**Kaempherol-*O*-dihexoside**	**1.72 ± 0.003**	**1.72 ± 0.003**
**10^e^**	17.29	324.00	193	MS^2^: 178(34), 134(100)	Ferulic Acid	0.14 ± 0.001	0.16 ± 0.003
**11^e^**	20.99	345.00	651	MS^2^: 609(65), 285(100)	Kaempherol-*O*-acetyl-dihexoside	0.53 ± 0.000	n.d.
					Total phenolic acids	1.04 ± 0.006	0.79 ± 0.005
					Total flavonoids	13.72 ± 0.019	10.85 ± 0.004
					Total Phenolic compounds	14.77 ± 0.013	11.64 ± 0.009

Rt: Retention time in minutes; λ_max_: wavelength (nm) of maximum absorption in the UV–visible region; [M-H]^−^: deprotonated ion (negative ion mode) (*m/z*); MS^n^ fragment ions generated in MS^2^ and/or MS^3^ spectra (*m/z*) and relative abundance in brackets, n.d.: not detected. Standard calibration curves used for quantification: apigenin-7-*O*-glucoside (*y* = 10.683*x* − 45.794; *R^2^* = 0.999, LOD (limit of detection) = 0.10 μg/mL; and LOQ (limit of quantification) = 0.53 μg/mL, peak 7); ferulic acid (*y* = 633.126*x* − 185.462, *R*^2^ = 0.999, LOD = 0.20 μg/mL; and LOQ = 1.01 μg/mL, peak 4, 6 and 10); and quercetin-3-*O*-glucoside (*y* = 34.843*x* − 160.173, *R^2^* = 0.9998, LOD = 0.21 µg/mL; LOQ = 0.71 µg/mL, peaks 1–3, 5, 8, 9, and 11).

**Table 3:** Peaks, retention times (RT), wavelengths of maximum absorption in the visible region (λ_max_), mass spectral data, tentative identification, and quantification (mg/g extract) of the phenolic compounds present in the hydroalcoholic (EtOH 80%) and decoction (D) extracts of *R. induratus*. Major compounds identified are highlighted in bold and shaded in gray.

Peak	RT (min)	λ_max_ (nm)	[M-H]^−^ (*m/z*)	MS^n^ (*m/z*)	Tentative Identification	Quantification (mg/g Extract)	
						EtOH 80%	Decoction
**1^r^**	5.51	324.00	341	MS^2^: 179(100), 135(18)	Caffeic Acid Hexoside	0.33 ± 0.007	0.17 ± 0.006
**2^r^**	**5.73**	**345**	**577**	**MS^2^: 413(100), 293(32)**	**Rhamnosylvitexin**	**1.22 ± 0.019**	**0.83 ± 0.017**
**3^r^**	6.49	264,329	947	MS^2^: 785(100). MS^3^: 639(100), 315(78)	Isorhamnetin-*O*-caffeoyl-*O*-deoxyhexosyl-dihexoside Isomer I	0.62 ± 0.000	0.55 ± 0.000
**4^r^**	6.72	264,311	947	MS^2^: 785(100). MS^3^: 639(100), 315(78)	Isorhamnetin-*O*-caffeoyl-*O*-deoxyhexosyl-dihexoside Isomer II	0.63 ± 0.006	0.59 ± 0.001
**5^r^**	7.87	311	325	MS^2^: 163(29), 145(100), 119(17)	*cis p*-Coumaric Acid Hexoside	0.16 ± 0.004	0.11 ± 0.002
**6^r^**	8.32	311	325	MS^2^: 163(29), 145(100), 119(17)	*trans p*-Coumaric Acid Hexoside	0.16 ± 0.003	0.09 ± 0.001
**7^r^**	9.37	312	355	MS^2^: 193(100), 179(11), 149(78)	Ferulic Acid Hexoside Isomer I	0.71 ± 0.000	0.46 ± 0.001
**8^r^**	9.88	312	355	MS^2^: 193(100), 179(16), 149(61)	Ferulic Acid Hexoside Isomer II	0.22 ± 0.008	0.15 ± 0.004
**9^r^**	14	340	563	MS^2^: 545(5), 503(6), 473(78), 443(100)	Apigenin 6-*C*-hexosyl-8-*C*-pentoside	0.20 ± 0.008	0.09 ± 0.017
**10^r^**	**14.71**	**348**	**579**	**MS^2^: 459(5), 429(71), 357(54), 327(100), 285(5)**	**Luteolin-6-*C*-(2-*O*-pentosyl)hexoside Isomer I**	**4.15 ± 0.053**	**2.64 ± 0.029**
**11^r^**	**15**	**345**	**579**	**MS^2^: 459(5), 429(65), 357(56), 327(100), 285(5)**	**Luteolin-6-*C*-(2-*O*-pentosyl)hexoside Isomer II**	**1.69 ± 0.040**	**0.96 ± 0.047**
**12^r^**	**16.55**	**336**	**563**	**MS^2^: 443(5), 413(100), 293(13)**	**Apigenin-*C*-hexoside-*O*-pentoside Isomer I**	**1.21 ± 0.002**	**0.72 ± 0.007**
**13^r^**	17.33	336	563	MS^2^: 443(5), 413(100), 293(13)	Apigenin-*C*-hexoside-*O*-pentoside Isomer II	0.18 ± 0.005	0.10 ± 0.007
**14^r^**	17.95	351	609	MS^2^: 301(100)	Quercetin-3-*O*-rutinoside	0.53 ± 0.002	0.50 ± 0.001
**15^r^**	18.56	335	431	MS^2^: 341(22), 311(100). MS^3^: 283(100)	Apigenin-*C*-hexoside	0.14 ± 0.009	0.07 ± 0.004
**16^r^**	18.87	355	639	MS^2^: 315(100)	Isorhamnetin-*O*-dihexoside Isomer I	0.50 ± 0.001	0.48 ± 0.000
**17^r^**	22.21	354	623	MS^2^: 315(100)	Isorhamnetin-*O*-dihexoside Isomer II	0.52 ± 0.000	0.50 ± 0.001
					Total Phenolic Acids	1.59 ± 0.007	0.99 ± 0.005
					Total Flavonoids	11.58 ± 0.123	8.03 ± 0.107
					Total Phenolic Compounds	13.18 ± 0.116	9.02 ± 0.112

Rt: Retention time in minutes; λ_max_: wavelength (nm) of maximum absorption in the UV–visible region; [M-H]^−^: deprotonated ion (negative ion mode) (*m/z*); MS^n^ fragment ions generated in MS^2^ and/or MS^3^ spectra (*m/z*) and relative abundance in brackets. Standard calibration curves used for quantification: apigenin-7-*O*-glucoside (*y* = 10.683*x* − 45.794; *R^2^* = 0.999, LOD (limit of detection) = 0.10 μg/mL; and LOQ (limit of quantification) = 0.53 μg/mL, peak 2); apigenin-6-*C*-glucoside (*y* = 107.025*x* + 61.531, *R^2^* = 0.999, LOD = 0.19 µg/mL; LOQ = 0.63 µg/mL, 9–13 and 15); caffeic acid (*y* = 388.345*x* + 406.369, *R^2^* = 0.994, LOD = 0.78 μg/mL; LOQ =1.97 μg/mL, peak 1); ferulic acid (*y* = 633.126*x* − 185.462, *R^2^* = 0.999, LOD = 0.20 μg/mL; and LOQ = 1.01 μg/mL, peak 7 and 8); *p*-coumaric acid (*y* = 301.950*x* + 6966.7, *R^2^* = 0.999, LOD = 0.68 μg/mL and LOQ = 1.61 μg/mL, peaks 5 and 6); and quercetin-3-*O*-glucoside (*y* = 34.843*x* − 160.173, *R^2^* = 0.999, LOD = 0.21 µg/mL; LOQ = 0.71 µg/mL, peaks 3, 4, 14, 16, and 17).

## Data Availability

All data generated or analyzed during this study are included in this published article.

## Supplementary Materials

The following supporting information can be downloaded at https://www.mdpi.com/article/10.3390/ph18010039/s1. Figure S1: Effect of silibinin, the key component of silymarin from *Silybum marianum* (L.) Gaertn., on HepG2 cells.
