# Supplementary material for: Chemical Characterization and Differential Lipid-Modulating Effects of Selected Plant Extracts from Côa Valley (Portugal) in a Cell Model for Liver Steatosis
Source: Pharmaceuticals (Basel). 2025 Jan 1;18(1):39. doi: 10.3390/ph18010039 (PMC11768118; doi:10.3390/ph18010039)
Supplement: Supplementary file 1 [file pharmaceuticals-18-00039-s001.zip › pharmaceuticals-3392768-supplementary.pdf]

SUPPLEMENTARY INFORMATION FILE

Chemical characterization and prevention of lipid accumulation in a cell model for Liver Steatosis by selected plant extracts from Côa Valley (Portugal)

Ricardo Amorim[1,2$], Mário Pedro Marques[1,2], Catarina Melim[1,2], Carla Varela[1,2,3], Vilma A. Sardão[2,4,5], José Teixeira[2,5], Maria Inês Dias[6,7], Lillian Barros[6,7], Paulo J. Oliveira[2,5], Célia Cabral[1,2,8*]

[1]University of Coimbra, Coimbra Institute for Clinical and Biomedical Research (iCBR), Clinic Academic Center of Coimbra (CACC), Faculty of Medicine, 3000-548 Coimbra, Portugal

[2]University of Coimbra, Center for Innovative Biomedicine and Biotechnology (CIBB), 3000-548 Coimbra, Portugal

[3]University of Coimbra, Chemical Engineering and Renewable Resources for Sustainability (CERES), Faculty of Sciences and Technology, 3030-790 Coimbra, Portugal

[4] Multidisciplinary Institute of Aging, MIA-Portugal

[5]University of Coimbra, CNC-UC, Center for Neuroscience and Cell Biology, 3004-504 Coimbra, Portugal

[6]Centro de Investigação de Montanha (CIMO), Instituto Politécnico de Bragança, Campus de Santa Apolónia, 5300-253 Bragança, Portugal

[7]Laboratório Associado para a Sustentabilidade e Tecnologia em Regiões de Montanha (SusTEC), Instituto Politécnico de Bragança, Campus de Santa Apolónia, 5300-253 Bragança, Portugal.

[8]Center for Functional Ecology, Department of Life Sciences, University of Coimbra, Calçada Martim de Freitas, 3000-456 Coimbra, Portugal

[$]**current address:** i3S – Institute for Research and Innovation in Health, Porto, Portugal

*Corresponding author:

Célia Cabral; Faculty of Medicine, University of Coimbra, Pólo das Ciências da Saúde, Azinhaga de Santa Comba, 3000-548 Coimbra, Portugal; Tel.: +351 239 480 066; E-mail address: celia.cabral@fmed.uc.pt

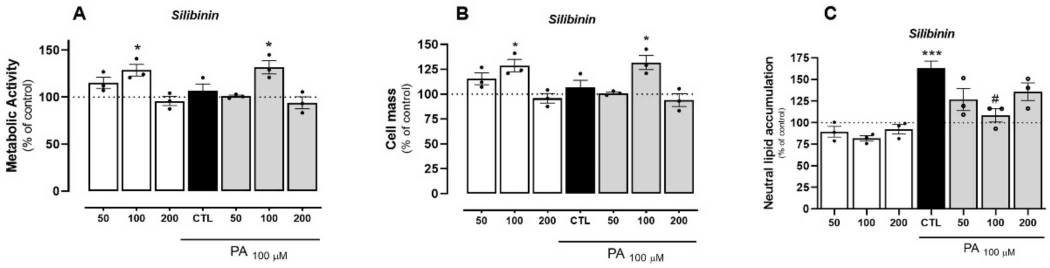

Fig. S1 Effect of silibinin, the key component of silymarin from *Silybum marianum* (L.) Gaertn., on HepG2 cells. (A)The metabolic activity, (B) cell mass, and (C) neutral lipid accumulation of HepG2 cells, in percentage of control, following silibinin incubation in three different concentrations (50, 100 and 200 µM), for 24h. B - The cell mass of HepG2 cells, in percentage of control, following silibinin incubation in three different concentrations (50, 100 and 200 µM). Each graph contains the results for silibinin incubation (white bars) and for PA following silibinin preincubation (grey bars). The black bar represents PA at 100 µM without extract preincubation, for comparison purposes. Statistical significance was compared using two-way ANOVA followed by Tukey's post hoc test for multiple comparisons (*$p<0.05$, ***$p<0.0005$ *vs* untreated cells); ([#]$p<0.05$, [##]$p<0.01$ *vs* PA-treated cells).
